# The Role of Doctor Visits, Body Image Discrepancy, and Perceived Health in Predicting Medical Weight Problem Diagnosis

**DOI:** 10.3390/healthcare13172135

**Published:** 2025-08-27

**Authors:** Norma Olvera, Rhonda Scherer, Weiwei Wu, Tamal J. Roy, Molly R. Matthews-Ewald, Weihua Fan, Consuelo Arbona

**Affiliations:** 1 Department of Psychological, Health & Learning Sciences, University of Houston, 3657 Cullen Boulevard, Farish Hall Room 491, Houston, TX 77204, USA; rlschere@central.uh.edu (R.S.); wwu13@cougarnet.uh.edu (W.W.); tjroy@cougarnet.uh.edu (T.J.R.); wfan@central.uh.edu (W.F.); consueloa@central.uh.edu (C.A.); 2 WhitworthKee Consulting, Washington, DC 20003, USA; mmathewsewald@whitworthkee.com

**Keywords:** Hispanic, men, women, body image, health status, healthcare, weight problem

## Abstract

**Background/Objectives**: This study investigated how doctor visit(s), body image discrepancy, and perceived health status are associated with receiving a medical weight problem diagnosis. **Methods**: The sample included 458 Hispanic adults (366 women, 92 men) who completed a health survey at health fairs. **Results**: Descriptive analyses indicated that 51.4% of women and 54.3% of men were classified as overweight or obese, yet only 30% received a medical weight problem diagnosis. Most participants selected an ideal body shape that was thinner than their perceived body shape. Separate logistic regression analyses were conducted by gender to assess associations between body image discrepancy, perceived health status, and receiving a medical weight problem diagnosis, controlling for age. Findings revealed that women who had visited a doctor in the past year had 5.02 times the odds (95% CI:1.98–12.73) of receiving a medical weight problem diagnosis compared to those who had not. Each one-point increase in body image discrepancy was associated with a 1.88-fold increase in the odds of receiving a diagnosis (95% CI:1.49–2.37). Conversely, a one-point increase in perceived health status was associated with 1.59 times the odds (95% CI: 0.47–0.83) of not receiving a diagnosis. For men, those who had visited the doctor in the past year had 14.17 times the odds (95% CI:1.53–131.17) of receiving a medical weight problem diagnosis. Each one-point increase in body image discrepancy was associated with 1.60 times the odds of receiving a diagnosis (95% CI:1.01–2.54). However, perceived health status was not a significant predictor of diagnosis among men. **Conclusions**: Addressing healthcare access barriers and considering the roles of body image and perceived health status could improve obesity diagnosis and treatment in Hispanic populations.

## 1. Introduction

The United States (U.S.) is experiencing an unprecedented rise in obesity rates, with projections indicating that by 2030, 78% of adults may be classified as overweight or obese [1]. This alarming trend has profound implications for healthcare costs, with obesity-related medical expenses estimated to exceed USD 173 billion annually in the U.S. [2]. These medical expenses are the result of comorbidities associated with obesity such as, but not limited to, hypertension, dyslipidemia, depression, anxiety, prediabetes, type 2 diabetes, coronary heart disease, and sleep apnea [3]. Of particular concern is the disproportionate impact on Hispanic adults, who consistently have higher obesity rates (45%) compared to their White (42%) and Asian (17%) counterparts [4]. This disparity highlights the pressing need for culturally tailored interventions and improved access to healthcare for Hispanic communities. Moreover, enhancing recognition of excess weight and self-efficacy in weight management among Hispanic adults is critical.

It should also be noted that Hispanic populations face unique barriers to healthcare, including lack of transportation [5], limited availability of Spanish-speaking providers, and communication difficulties with their providers [6]. These factors contribute to lower rates of health screenings and fewer opportunities for weight-related discussions with healthcare providers. Despite this, considerable variability exists in healthcare access and utilization within Hispanic communities [7], further underscoring the importance of understanding how to best support these populations.

### 1.1. Importance of Medical Diagnosis of Obesity

Healthcare utilization refers to the use of all types of healthcare services to prevent and cure health problems [8], including doctor visit(s) where an ambulatory patient is seeking care from a licensed physician [9]. Receiving a medical diagnosis of obesity (defined as an excessive accumulation of fat presenting a health risk to the individual [10] is a crucial first step toward effective weight management [11]. Further, patients are more likely to adopt and sustain healthy lifestyle changes, such as improved diet and increased physical activity, when guided by a trusted healthcare provider [12,13]. For instance, a study of 14,256 patients enrolled in a weight loss program found that those with more frequent doctor visits maintained a greater than 10% weight loss over 12 months compared to those with fewer visits [14]. Maintaining this level of weight loss has significant individual and public health-level impacts. Even a modest 5% weight loss has been linked to improvements in obesity-related comorbidities when initiated through a medical diagnosis by a physician [15,16]. Despite these benefits, the rate of medical obesity diagnosis has declined significantly, dropping from 33% in 2008–2009 to 21% in 2012–2013 [17]. This decline persists despite the increasing prevalence of obesity and highlights the need for more consistent and proactive identification of obesity in clinical settings. Research shows that only 55% of patients with obesity received a diagnosis, and among those diagnosed, a mere 24% scheduled follow-up appointments [18].

Hispanic adults are particularly affected, as they receive fewer health screenings than White and Asian adults, further reducing their likelihood of receiving an obesity diagnosis and appropriate weight management counseling [9,19]. For instance, Nguyen and colleagues [20] found that 52% of Mexican American women and 53% of men with obesity had never been advised by a doctor to reduce intake of high-fat or high-cholesterol foods. Yet Lewis et al. [21] reported that Hispanic and Black adults with obesity desired more weight control counseling from their healthcare providers. Together, these findings underscore the importance of providing timely medical diagnosis and subsequent referrals for weight management in Hispanic patients.

### 1.2. Body Image Discrepancy

Body image discrepancy (BID), or the difference between one’s perceived body size and ideal body size, may significantly influence whether individuals seek a weight diagnosis and weight control advice. BID affects both women and men across various body sizes [22]. Research indicates that as body size increases, so does the desire to be thinner, regardless of ethnicity [23]. However, BID manifests differently for women and men. Women generally report higher BID as BMI increases, whereas men tend to show significant BID usually when BMI reaches 30 kg/m^2^ or higher [22]. This complex relationship affects health-seeking behaviors in contradictory ways. For example, Gruszka et al. [24] found that women who were dissatisfied with their body size were more likely to seek medical help, while Cook et al. [25] noted that greater BID in women sometimes led to avoidance of doctor visits to prevent being weighed. Similarly, Austin et al. [26] found that higher body dissatisfaction reduced adherence to weight loss treatments. Despite these findings, no studies to date have specifically examined how BID influences medical weight problem diagnosis or weight control treatment among Hispanic women and men.

### 1.3. Perceived Health Status

Perceived health status, or an individual’s self-evaluation of their overall physical well-being, is another central psychosocial factor influencing medical consultation [27]. Perceived health status is grounded in both theoretical models of health behavior and empirical evidence. Rosenstock’s Health Belief Model [28] uses an expectancy-value framework and posits that perceived susceptibility and perceived severity are key factors in health-related decision-making, including the likelihood of seeking medical care or initiating weight management behaviors [29]. Individuals who rate their health as poor may perceive themselves to be at greater risk for adverse outcomes associated with excess weight, thereby increasing their motivation to act.

Empirically, perceived health status has been consistently linked to health service utilization, obesity recognition, and treatment engagement. For example, Idema et al. [30] found that young adults who perceived themselves as overweight reported poorer general health and were more likely to acknowledge weight-related health risks compared to those who considered their weight healthy. Essayli et al. [31] also demonstrated that perceived health status moderated the impact of weight labels on body dissatisfaction and health perceptions in college-aged women. Thus, individuals who rate their health as poor are more likely to recognize health risks, receive obesity-related diagnoses, and engage in weight management treatment.

These well-established findings suggest that perceived health may act as a motivational factor for recognizing weight issues and, in turn, seeking (and later, engaging in) treatment. Prior research also underscores the importance of addressing health perceptions in promoting positive body image and weight loss behaviors. To our knowledge, only one study conducted by Reesor and colleagues [32] explored the relationship between body size, body image, and perceived health status in Hispanic women with overweight and obesity. They found that women who rated their health as poor had 84% lower odds of underestimating their weight, suggesting that negative health perceptions may improve body size awareness. However, Hispanic men have largely been absent from such studies, creating a critical gap in understanding how perceived health status influences obesity diagnosis across genders.

### 1.4. Purpose of Current Study

Despite the growing Hispanic population and rising obesity rates, little is known about how doctor visits, BID, and self-perceived health status relate to receiving medical obesity diagnosis among Hispanic women and men. This study aims to address this gap by examining these factors. Understanding these relationships is essential for developing tailored medical and community interventions for Hispanic adults. For the current study, it was hypothesized that self-perceived health status, BID, and having visited a doctor in the past 12 months would be positively associated with receiving a medical obesity diagnosis among Hispanic women and men.

## 2. Materials and Methods

### 2.1. Participants

This study included a sample of 458 Hispanic adults (366 women and 92 men) as part of a larger study examining the roles of medical obesity diagnosis, medical weight control advice, perceived body image, and perceived health status among Hispanic and African American adults. The larger study inclusion criteria required participants to be (a) a Hispanic or African American adult (women or men); (b) 18 years or older; and (c) able to read in English or Spanish. For the purposes of the present study, only data from Hispanic women and men were analyzed. The mean age of the sample was 41.2 years (*SD* = 13.7), with women averaging 40.3 years (*SD* = 13.2) and men averaging 43.4 years (*SD* = 14.6). All study protocols were approved by the University’s Institutional Review Board (reference ID #00000671).

### 2.2. Procedures

Detailed descriptions of the study procedures and measures are available in Olvera and colleagues [33]. Briefly, eligible participants were recruited at four large community health fairs held in predominantly Hispanic and African American neighborhoods between 2017 and At each health fair, researchers staffed a table displaying nutritional information and other health resources for attendees. After individuals visited the table and received health resources, they were invited to learn about the study. Interested individuals were informed about the study’s purpose, assessments, and estimated completion time (10–15 min). Those who agreed to participate signed an informed consent form and then completed an anonymous health survey and the Stunkard Figure Rating Scale (SFRS) [34] in English or Spanish. Bilingual research assistants were available to answer any questions about the study’s instruments or procedures. Upon completion, participants received a t-shirt as an appreciation for their participation. Response rates ranged from 80% to 90%, with an average of 85%.

### 2.3. Measures

#### 2.3.1. Health Survey

The health survey collected the following information: (1) demographic characteristics (e.g., age, gender, and ethnicity); (2) self-reported weight and height, which were used to calculate participants’ body mass index (BMI); (3) self-reported general health (“*In general, how would you rate your current health?*”) measured on a 5-point Likert-type scale (1 = *very poor* to 5 = *very good*); (4) whether the participant had visited a doctor in the past 12 months (“*Yes*” *or* “*No*”), followed by a question asking if the participant sought medical consultations in the past 12 months for health issues such as *sick visits* (e.g., *cold*, *flu*, *coughing*, *fever*), *chronic conditions* (e.g., *diabetes*, *high blood pressure*, *asthma*), *physical/check-ups*, *skin disorders* (e.g., *acne*, *dermatitis*), *joint disorders* (e.g., *arthritis*), *back problems*, *mood disorders* (e.g., *anxiety*, *depression*), *headaches and migraines, and weight problem*; and (5) whether the participant had ever received a medical weight problem diagnosis (“*Has your doctor ever told you that you have a weight problem?*”) with “Yes” or “No” response options.

#### 2.3.2. Stunkard Figure Rating Scale (SFRS) [34]

The SFRS [34] is a pictorial assessment tool used to assess perceived and ideal body size, as well as BID. It consists of nine gender-specific silhouettes, ranging from *very thin* (corresponding value of 1) to *very obese* (corresponding value of 9). To assess perceived body size, participants responded to “*How do you think you look?*” by selecting the silhouette that best represented their current appearance. For ideal body size, participants selected the silhouette corresponding to “*The way you would like to look.*” BID was calculated as the difference between perceived body size and ideal body size, with positive value indicating a desire to be thinner and negative values indicating a desire to be heavier.

The SFRS has demonstrated good test–retest reliability and has shown positive correlations with drive for thinness (*r* = 0.85) and BID (*r* = 0.91) [35]. The instrument has been shown to be a reliable and valid instrument for Hispanic adults. Specifically, Meza Peña et al. [36] reported a positive association between SFRS-BID and BMI (*r* = 0.23, *p* < 0.001) among ethnically diverse populations, including Hispanic adults. Additionally, Martínez-Aldao et al. [37] found that the SFRS was reliable among Spanish adults (*ICC* = 0.80) and that SFRS-perceived body size was correlated with BMI (*r* = 0.70). In the current study, perceived body size ratings were significantly correlated with BMI for both women (*r* = 0.72, *p* < 0.001) and men (*r* = 0.49, *p* < 0.001).

### 2.4. Statistical Analysis

Descriptive statistics, including means and standard deviations for continuous variables and percentages for categorical variables, were computed to characterize the sample. Participants’ BMI was calculated from self-reported weight and height using the standard formula [weight in kg/height^2^ (meters)]. BMI categories were determined according to the CDC adult classifications: underweight (<18.5 kg/m^2^), healthy weight (18.5 kg/m^2^ to 24.9 kg/m^2^), overweight (25.0 kg/m^2^ to 29.9 kg/m^2^), and obesity (≥30 kg/m^2^) [38]. Previous research has shown that self-reported weight and height are highly correlated with objectively measured height and weight values (*r* = 0.84 to 0.92) [39]; (*r* = 0.87 to 0.92) [40].

To test the study hypothesis, separate logistic regression analyses were conducted for Hispanic men and women to identify factors associated with the likelihood of receiving a weight diagnosis from a medical professional, controlling for age. Cases with missing data on any predictor or outcome variables were excluded using listwise deletion, the default procedure in IBM SPSS Statistics. Self-perceived health, BID, and having had at least one doctor visit in the past 12 months were entered stepwise into the models with age as the control variable. Age was included as a control variable given that body dissatisfaction remains relatively stable throughout adulthood [41]. All analyses were conducted using IBM SPSS Statistics version 29.0, with statistical significance set at *p <*  0.05.

## 3. Results

### 3.1. Sample Descriptive Characteristics

As shown in Table 1, almost 75% of participants rated their health status as average or good. Most participants (83.1% women, 80.4% men) reported visiting a doctor in the past 12 months. About 30% of the sample (31.7% women, 28.3% men) reported receiving a weight problem diagnosis from a medical professional. Notably, 18.3% of women and 28.3% of the men did not answer the question about doctor visit(s) in the past 12 months.

Self-reported mean height for women was 63.0 inches (*SD* = 3.1 inches) and 66.3 inches (*SD* = 4.0 inches) for men. Self-reported mean weight was 161.6 pounds (*SD* = 35.0 pounds) for women and 175.8 pounds (*SD* = 34.7 pounds) for men (not shown in Table 1). Among participants who provided height and weight data, slightly over 50% were classified as either with overweight or obesity (Table 1).

Among women who reported medical consultation in the past 12 months, reasons included the following: physicals/checkups (48%), sickness (28%), chronic conditions (18%), headaches and migraines (22%), joint disorders (10%), back problems (14%), skin disorders (11%), mood disorders (11%), and weight problems (4%). Men sought medical consultations for the following: physicals/checkups (39%), sickness (23%), chronic conditions (22%), headaches and migraines (14%), joint disorders (10%), back problems (13%), skin disorders (7%), mood disorders (5%), and weight problems (2%).

It is also noteworthy that over one-third of both women and the men did not know their weight and/or height; consequently, their BMI could not be calculated. When examining differences in the proportions of women and men visiting a doctor in the last 12 months, a chi-square test of independence revealed no statistical significance [χ^2^(1, 449) = 0.705, *p* = 0.401]. Similarly, no significant difference was found in the proportion of women and men receiving a medical weight problem diagnosis [χ^2^(1, 365) = 0.008, *p* = 0.928]. These indicate no gender differences in either visiting a doctor in the prior 12 months or receiving a weight problem diagnosis. In addition, χ^2^ tests of independence examined whether non-responders differed from responders by gender and age across obesity status and weight problem diagnosis. No differences between non-responders and responders were found regarding obesity status by gender (χ^2^(1) = 0.43, *p* = 0.512) or by age (χ^2^ (1) = 0.68, *p* = 0.410). Similarly, for medical weight problem diagnosis, there was no difference between responders and non-responders by age (χ^2^(1) = 0.51, *p* = 0.475). However, there was a significant difference in receiving a medical weight problem diagnosis by gender, with men being more likely than women to skip this question (χ^2^(1) = 4.50, *p <* 0.05).

### 3.2. Perceived and Ideal Body Size and Body Image Discrepancy

**Figure 1:**
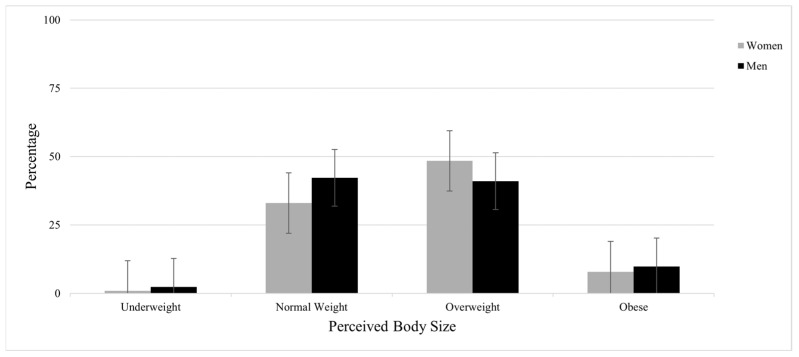
Distribution of participant perceived body size by gender (women: *n* = 337; men: *n* = 83).

As shown in Figure 1, a small percentage of participants (0.9% women, 2.4% men) selected the underweight body size silhouette as their perceived body size. For normal silhouettes, 33% of women and 42.2% of men selected these as representative of their body size. Nearly half of women (48.4%) and 41% of men selected the overweight silhouette as representative of their body size, while 17.9% of women and 14.4% of men selected the obese silhouettes as representative of their body size. Notably, 7.9% of women and 9.8% of men did not answer this question.

**Figure 2:**
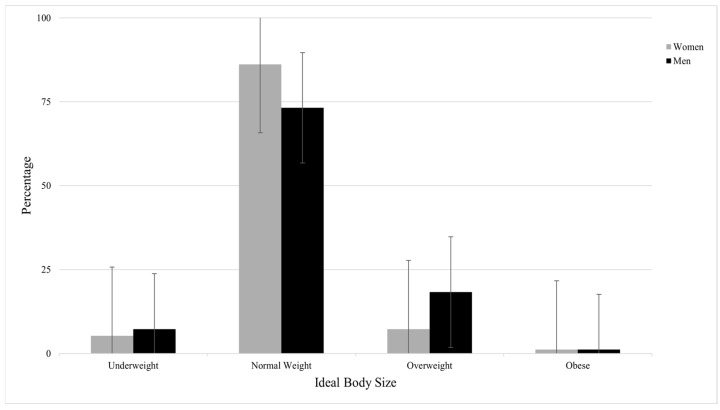
Distribution of participant selection of ideal body size by gender (women: *n* = 342; men: *n* = 82).

Regarding the selection of their ideal body size selection, Figure 2 illustrates that 5.3% of women and 7.3% of men endorsed the underweight silhouette as their ideal body size. The majority of participants selected normal weight silhouettes as ideal (86.2% of women, 73.2% of men), and 7.3% of women and 18.3% of men endorsed the overweight silhouette as their ideal body size. Only 1.2% of both women and men endorsed the obese silhouettes as ideal.

**Figure 3:**
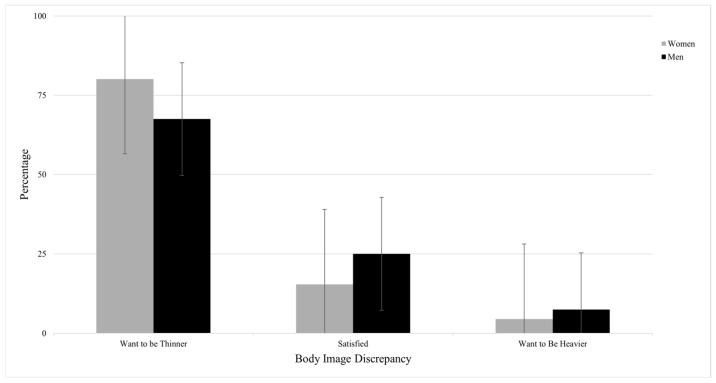
Distribution of participant body image discrepancy by gender (women: *n* = 342; men: *n* = 80).

Lastly, as shown in Figure 3, only 15.4% of the women and 25.0% of the men selected ideal body sizes that were aligned with their perceived actual body size. In contrast, a high percentage of participants desired to be thinner (80.1% of the women, 67.5% of men), while a small percentage desired to be heavier (4.5% of women, 7.5% of men). Body size discrepancy could not be calculated for some participants (9.6% women, 13% men) because of missing either their perceived or ideal body size selection, or both.

### 3.3. Logistic Regression Analysis to Predict Medical Weight Problem Diagnosis

Two separate logistic regressions were conducted to investigate how different variables were associated with medical weight problem diagnosis for women and men after controlling for age. As shown in Table 2, the separate logistic regression analyses for women and men included doctor visit(s) during the past 12 months, perceived health status, and BID as predictors, with age as a control variable.

Results indicated that the full model was statistically significant *(χ*^2^ *=* 71.39, *df* = 4, *p* < 0.001) and explained 22.6% (Cox & Snell R^2^) to 30.7% (Nagelkerke R^2^) of the variance in weight problem diagnosis and correctly classified 72.7% of cases. At least one doctor visit during the past 12 months *(OR =* 5.02, 95% CI: 1.98–12.73), perceived health status (*OR =* 0.63, 95% CI: 0.47–0.83), and BID (*OR *= 1.88, 95% CI: 1.49–2.37) all significantly predicted women’s likelihood of reporting the receipt of a medical weight problem diagnosis, controlling for age. Hispanic women who visited the doctor in the past 12 months were 5.02 times as likely to receive a medical weight problem diagnosis as those who had not. That is, a one-point increase in BID was associated with 1.88 the odds of obtaining a medical weight problem diagnosis for Hispanic women. Inverting the odds ratio reveals that a one-point increase on perceived health status was associated with 1.59 times the odds of not obtaining a medical weight diagnosis.

The full logistic regression analysis for men (Table 2) was also statistically significant *(χ^2^ =* 15.784, *df* = 4, *p* < 0.05) and explained 24.2% (Cox & Snell R^2^) to 32.8% (Nagelkerke R^2^) of the variance in weight problem diagnosis and correctly classified 77.2% of cases. Significant predictors included at least one doctor visit during the past 12 months *(OR =* 14.17, 95% CI: 1.53–131.17) and BID (*OR =* 1.60, 95% CI: 1.01–2.54), controlling for age. Hispanic men who visited a doctor at least once in the past 12 months were 14.17 times as likely to report receiving a medical weight problem diagnosis as those who had not. That is, a one-point increase in BID was associated with the 1.60 times the odds of obtaining a medical weight problem diagnosis for Hispanic men. Unlike women, perceived health status was not a significant predictor of medical weight problem diagnosis for men (*OR *= 0.74, 95% CI: 0.45–1.23), suggesting that Hispanic men’s perceived health status did not influence their likelihood of receiving medical weight problem diagnosis.

## 4. Discussion

The purpose of this study was to examine the association of BID, perceived health status, and doctor visit(s) in the past 12 months with the probability of receiving a medical weight problem diagnosis among Hispanic women and men. Descriptive analyses indicated that, based on participant self-reported height and weight, slightly over half of the sample was classified as either overweight or obese. Despite this, a very few participants reported visiting a doctor for a weight problem in the past 12 months. Instead, most participants reported visiting a doctor for physical/checkups, sickness, and chronic conditions. In addition, only 30.0% received a weight problem diagnosis from a medical professional. It is noteworthy that approximately 35% of all participants did not know their height or weight; therefore, BMI and obesity status could not be calculated for these individuals.

The current study offers valuable insights into factors associated with medical weight problem diagnosis among Hispanic women and men. BID and at least one doctor visit in the past 12 months were significantly associated with receiving a medical weight problem diagnosis for both Hispanic women and men. These findings suggest that participants who were more dissatisfied with their body size and visited a doctor in the past 12 months had increased odds of receiving a medical weight problem than those who were more satisfied with their body size and did not visit a doctor in the previous year.

This study also revealed that self-perceived poor health status was associated with increased odds of receiving a medical weight problem diagnosis, but only for women. This indicates potential gender differences in how health perceptions influence weight-related discussions between patients and healthcare providers. The observed gender differences in factors associated with weight problem diagnosis suggest that interventions may need to be tailored differently for Hispanic women and men.

Findings from this study have several important implications for healthcare practice. First, the relatively low rate of weight problem diagnosis when compared to classification of the participants based on self-reported height and weight suggests a need for additional awareness and training among healthcare providers to address weight issues more consistently during patient visits. This pattern is consistent with other studies. Fitzpatrick and colleagues [17] reported that less than 30% of patients with obesity in their sample received a formal obesity diagnosis during primary care visits. Similarly, Pantalone and colleagues [11] found that only 48% of patients classified as obese received a formal obesity diagnosis, while Kaplan and colleagues [18] reported that among approximately 3000 patients with obesity, only 55% received an obesity diagnosis. These findings are particularly concerning for Hispanic populations. Alemán and colleagues [7] that indicated that Hispanic individuals with overweight and obesity receive fewer health screenings than those with normal weight. Therefore, efforts to reduce barriers to healthcare access for Hispanic adults should be prioritized to increase opportunities for weight-related discussions and interventions.

Healthcare providers may particularly benefit from cultural competence training to better understand and address the unique needs and perspectives of Hispanic patients regarding BID, weight, and perceived health status. This training would align with findings from Lewis and colleagues [21] who found that Hispanic and African American adults with obesity expressed a desire to receive more weight control counseling from their healthcare providers.

Moreover, the findings from this study support the development and evaluation of culturally tailored interventions that address the unique needs and perspectives of Hispanic adults regarding body image and perceived health status. This includes considerations such as primary language(s) spoken and cultural norms regarding factors such as body shape and eating. Doing so would support the assertion of the study conducted by Rojas-Guyler and colleagues [42] that there is a need for novel interventions aimed at reducing health care disparities targeting Hispanic populations.

Given the association between BID and weight problem diagnosis in Hispanic women and men, incorporating body image assessments into routine healthcare visits may help identify individuals who may be ready for weight management interventions. However, providers should exercise caution to ensure that BID is not associated with other psychological factors or does not lead to unhealthy weight control practices.

Although this study provides valuable insights, it is important to acknowledge its several limitations. First, it was not possible to collect exhaustive participant demographic information (e.g., health insurance status) to determine how well this sample represents the broader Hispanic adult populations. Second, over 30% of the study samples have missing weight or height data, and about 23% of participants did not report if they have received a weight problem diagnosis.

These findings are revealing but not surprising, as they align with previous research. For example, the Mexican Health and Aging Study found that 31.7% Hispanic adults did not self-report their height and 13.4% did not self-report their weight [43]. Similarly, according to the 2006 Mexican National Health and Nutrition Survey, only 20.2% of Hispanic adults with obesity received an obesity diagnosis from a health professional [44]. Several reasons may explain the missing height, weight, and medical obesity diagnosis: (1) infrequent visits to the doctor, which reduce opportunities to be weighed, have height measured, and receive information about obesity status; (2) discomfort and avoidance behaviors, where discomfort with body size leads individuals to avoid asking direct questions about their weight and obesity status—a pattern consistent with research showing that women with greater BID sometimes avoid doctor visits specifically to prevent being weighed [45]; and (3) healthcare disengagement where missing data may reflect wider patterns of healthcare avoidance among those in this population. The failure to receive or recall a formal medical diagnosis could be intertwined with documented difficulties Hispanic populations face in accessing and communicating with healthcare providers, including lack of health insurance and limited English proficiency.

Another potential limitation of this study is the non-responder bias. While results showed that missingness on obesity status was not systematically related to gender, there was a statistically significant association between gender and the likelihood of not responding to the medical weight problem diagnosis question. Specifically, a larger proportion of men did not answer this question compared to women. This systematic difference indicates that the findings from the logistic regression analyses should be interpreted with caution, as they may be influenced by the non-response bias.

Additionally, the self-reported health data were collected during health fairs. Because of the setting, it is possible that the individuals who were in attendance were already focused on their health and may therefore have reported being more concerned about their weight than the general population.

A methodological limitation of this study is the use of the SFRS. While the utilization of the SFRS is appropriate in terms of reliability and construct validity, its cultural relevance for Hispanic adults, especially men, requires further examination. This scale was developed in a different cultural context, which might not align with the body norms of the Hispanic population. Future studies should examine the cultural validity of the SFRS among Hispanic adults of varied demographic characteristics.

Further, age was controlled in the logistic regression models rather than examined as an independent predictor, based on prior research (largely with White populations) indicating that body dissatisfaction remains relatively stable in adulthood [41]. However, it may be warranted to examine whether age impacts the outcomes of this study, given the positive association between BID and age in younger populations. Nevertheless, because the models controlled for age and BID and doctor visit were still associated with a medical weight diagnosis, the current findings imply associations are present regardless of age. In the future, studies should examine the moderating role of age on degree of BID.

Lastly, the cross-sectional nature of the data limits causal inferences, and the reliance on self-reported measures may introduce bias. Future research should further explore this topic by designing and implementing longitudinal studies to examine the long-term impact of medical weight problem diagnosis on weight management behaviors and outcomes among Hispanic adults.

## 5. Conclusions

This study illustrates the interplay of factors influencing medical weight problem diagnosis among Hispanic women and men. By addressing barriers to medical care, improving and expanding provider training, and considering the roles of body image and self-perceived health, interventions can be developed to enhance obesity diagnosis and treatment in this underserved population. The findings underscore the need for a multifaceted approach that combines individual-level interventions with systemic changes in healthcare delivery. It is crucial to develop culturally sensitive and gender-specific strategies that not only increase the frequency of accurate medical weight problem diagnosis but also provide comprehensive support for weight management among Hispanic women and men. Such culturally responsive strategies could include incorporating body image assessments into routine medical care or developing educational resources in English and Spanish that acknowledge cultural attitudes toward perceived and ideal body size and BID. By implementing approaches such as these, we can work towards reducing obesity rates, improving overall health outcomes, and addressing health disparities in this important and growing segment of the U.S. population.

## Figures and Tables

**Table 1:** Descriptive characteristics of Hispanic women and men (N = 458).

Variables	Women (*n* = 366)	Men (*n* = 92)
	*n* (%)	*n* (%)
Self-perceived Health Status		
Very Poor	4 (1.1)	3 (3.3)
Poor	18 (4.9)	3 (3.3)
Average	142 (38.8)	25 (27.2)
Good	130 (35.5)	41 (44.6)
Very Good	55 (15.0)	9 (9.8)
Not Sure	13 (3.6)	6 (6.5)
Did Not Answer	4 (1.1)	5 (5.4)
Doctor Visit past 12 Months		
Yes	304 (83.1)	74 (80.4)
No	54 (14.8)	17 (18.5)
Did Not Answer	8 (2.2)	1 (1.1)
Medical Weight Problem Diagnosis		
Yes	116 (31.7)	26 (28.3)
No	183 (50.0)	40 (43.5)
Did Not Answer	67 (18.3)	26 (28.3)
Obesity Status		
Underweight	0 (0.0)	0 (0.0)
Normal Weight	56 (15.3)	8 (8.7)
Overweight	79 (21.6)	30 (32.6)
Obese	109 (29.8)	20 (21.7)
Did Not know (weight or heigh)	122 (33.3)	34 (37.0)

**Table 2:** Logistic regression coefficients for Hispanic women and men.

					95% CI	
Participants	Variables	B	Wald	Exp(B)	LL	UL
Women	Age	−0.005	0.22	1.00	0.97	1.02
	Doctor Visit(s)	1.61 **	11.56	5.02	1.98	12.73
	Health Status	−0.47 **	10.97	0.63	0.47	0.83
	BID	0.63 **	28.55	1.88	1.49	2.37
Men	Age	0.01	0.13	1.01	0.97	1.05
	Doctor Visit(s)	2.65 *	5.45	14.17	1.53	131.17
	Health Status	−0.30	1.32	0.74	0.45	1.23
	BID	0.47 *	3.99	1.60	1.01	2.54

* *p* < 0.05, ** *p* < 0.001 Note. B = unstandardized regression coefficient; CI = confidence interval; LL = lower limit; UL = upper limit; Exp(B) = odds ratio; Doctor Visit(s): doctor visit(s) in last 12 months; Health Status: self-perceived health status; BID = body image discrepancy: (perceived–ideal body size).

## Data Availability

The data are not publicly available due to concerns regarding privacy.

## References

[1] Wang Y., Beydoun M.A., Min J., Xue H., Kaminsky L.A., Cheskin L.J. (2020). Has the Prevalence of Overweight, Obesity and Central Obesity Levelled off in the United States? Trends, Patterns, Disparities, and Future Projections for the Obesity Epidemic. Int. J. Epidemiol..

[2] Center for Disease Control (CDC) Overweight & Obesity. https://www.cdc.gov/obesity/basics/consequences.html/.

[3] Bae J.P., Nelson D.R., Boye K.S., Mather K.J. (2025). Prevalence of Complications and Comorbidities Associated with Obesity: A Health Insurance Claims Analysis. BMC Public Health.

[4] Center for Disease Control (CDC) Prevalence of Obesity and Severe Obesity Among Adults: United States 2017–2018. https://www.cdc.gov/nchs/products/databriefs/db360.htm.

[5] Wolfe M.K., McDonald N.C., Holmes G.M. (2020). Transportation Barriers to Health Care in the United States: Findings From the National Health Interview Survey, 1997–2017. Am. J. Public Health.

[6] Galvan T., Lill S., Garcini L.M. (2021). Another Brick in the Wall: Healthcare Access Difficulties and Their Implications for Undocumented Latino/a Immigrants. J. Immigr. Minor. Health.

[7] Alemán J.O., Almandoz J.P., Frias J.P., Galindo R.J. (2023). Obesity Among Latinx People in the United States: A review. Obesity.

[8] Gavina C., Borges A., Afonso-Silva M., Fortuna I., Canelas-Pais M., Amaral R., Costa I., Seabra D., Araújo F., Tavei-ra-Gomes T. (2024). Patients’ Health Care Resources Utilization and Costs Estimation Across Cardiovascular Risk Categories: Insights from the LATINO Study. Health Econ. Rev..

[9] Center for Disease Control (CDC) Ambulatory Care Use and Physician Office Visits. https://www.cdc.gov/nchs/fastats/physician-visits.htm.

[10] World Health Organization Obesity and Overweight. http://www.who.int/news-room/fact-sheets/detail/obesity-and-overweight.

[11] Pantalone K.M., Hobbs T.M., Chagin K.M., Kong S.X., Wells B.J., Kattan M.W., Bouchard J., Sakurada B., Milinovich A., Weng W. (2017). Prevalence and Recognition of Obesity and Its Associated Comorbidities: Cross-Sectional Analysis of Electronic Health Record Data from a Large US Integrated Health System. BMJ Open.

[12] Loprinzi P.D., Davis R.E. (2016). Promotion of Weight Loss by Health-Care Professionals: Implications for Influencing Weight Loss/Control Behaviors. Am. J. Health Promot..

[13] Mainous A.G., Xie Z., Dickmann S.B., Medley J.F., Hong Y.-R. (2023). Documentation and Treatment of Obesity in Primary Care Physician Office Visits: The Role of the Patient-Physician Relationship. J. Am. Board Fam. Med..

[14] Lenoir L., Maillot M., Guilbot A., Ritz P. (2015). Primary Care Weight Loss Maintenance with Behavioral Nutrition: An Observational Study. Obesity.

[15] Pool A.C., Kraschnewski J.L., Cover L.A., Lehman E.B., Stuckey H.L., Hwang K.O., Pollak K.I., Sciamanna C.N. (2014). The Impact of Physician Weight Discussion on Weight Loss in US Adults. Obes. Res. Clin. Pract..

[16] Williamson D.A., Bray G.A., Ryan D.H. (2015). Is 5% Weight Loss a Satisfactory Criterion to Define Clinically Significant Weight Loss?. Obesity.

[17] Fitzpatrick S.L., Stevens V.J. (2017). Adult Obesity Management in Primary Care, 2008–2013. Prev. Med..

[18] Kaplan L.M., Golden A., Jinnett K., Kolotkin R.L., Kyle T.K., Look M., Nadglowski J., O’Neil P.M., Parry T., Tomaszewski K.J. (2017). Perceptions of Barriers to Effective Obesity Care: Results from the National ACTION Study. Obesity.

[19] Byrd A.S., Toth A.T., Stanford F.C. (2018). Racial Disparities in Obesity Treatment. Curr. Obes. Rep..

[20] Nguyen H.T., Markides K.S., Winkleby M.A. (2011). Physician Advice on Exercise and Diet in a U.S. Sample of Obese Mexi-can-American Adults. Am. J. Health Promot..

[21] Lewis K.H., Gudzune K.A., Fischer H., Yamamoto A., Young D.R. (2016). Racial and Ethnic Minority Patients Report Different Weight-Related Care Experiences Than Non-Hispanic Whites. Prev. Med. Rep..

[22] Olson K.L., Lillis J., Panza E., Wing R.R., Quinn D.M., Puhl R.R. (2020). Body Shape Concerns Across Racial and Ethnic Groups Among Adults in the United States: More Similarities than Differences. Body Image.

[23] Weinberger N.-A., Kersting A., Riedel-Heller S.G., Luck-Sikorski C. (2016). Body Dissatisfaction in Individuals with Obesity Com-pared to Normal-Weight Individuals: A Systematic Review and Meta-Analysis. Obes. Facts.

[24] Gruszka W., Owczarek A.J., Glinianowicz M., Bąk-Sosnowska M., Chudek J., Olszanecka-Glinianowicz M. (2022). Perception of Body Size and Body Dissatisfaction in Adults. Sci. Rep..

[25] Cook M., Winter V.R., O’Neill E.A. (2020). Body Appreciation and Health Care Avoidance: A Brief Report. Health Soc. Work..

[26] Austin J.L., Serier K.N., Sarafin R.E., Smith J.E. (2017). Body Dissatisfaction Predicts Poor Behavioral Weight Loss Treatment Ad-herence in Overweight Mexican American Women. Body Image.

[27] Goldstein M.S., Siegel J.M., Boyer R. (1984). Predicting Changes in Perceived Health Status. Am. J. Public Health.

[28] Rosenstock I. (1990). The Health Belief Model: Explaining Health Behavior through Expectancies. Health Behav. Health Educ. Theory Res. Pract..

[29] Faghih M., Kaveh M.H., Nazari M., Khademi K., Hasanzadeh J. (2024). Effect of Health Belief Model-Based Training and Social Support on the Physical Activity of Overweight Middle-Aged Women: A Randomized Controlled Trial. Front. Public Health.

[30] Idema C.L., Roth S.E., Upchurch D.M. (2019). Weight Perception and Perceived Attractiveness Associated with Self-Rated Health in Young Adults. Prev. Med..

[31] Essayli J.H., Murakami J.M., Wilson R.E., Latner J.D. (2016). The Impact of Weight Labels on Body Image, Internalized Weight Stigma, Affect, Perceived Health, and Intended Weight Loss Behaviors in Normal-Weight and Overweight College Women. Am. J. Health Promot..

[32] Reesor L., Canales S., Alonso Y., Kamdar N.P., Hernandez D.C. (2018). Self-Reported Health Predicts Hispanic Women’s Weight Perceptions and Concerns. Am. J. Health Behav..

[33] Olvera N., Matthews-Ewald M., Zhang R., Scherer R., Fan W., Arbona C. (2023). Weight Concern and Body Image Dissatisfaction among Hispanic and African American Women. Women.

[34] Stunkard A.J., Sørensen T., Schulsinger F. (1983). Use of the Danish Adoption Register for the study of Obesity and Thinness. Res. Publ. Assoc. Res. Nerv. Ment. Dis..

[35] Thompson J.K., Altabe M.N. (1991). Psychometric Qualities of the Figure Rating Scale. Int. J. Eat. Disord..

[36] Peña C.M., Guajardo E.P. (2018). Body Image Distortion and Dissatisfaction in a mexican sample. Rev. Psicol. Cienc. Comport. Unidad acad. Cienc. Juríd. Soc..

[37] Martínez-Aldao D., Diz J., Varela S., Sanchez-Lastra M.A., Pérez C.A. (2021). Reliability and validity of the SAPF questionnaire and the Stunkard rating scale amongst elderly Spanish people. An. Sist. Sanit. Navar..

[38] CDC Adult BMI Categories. https://www.cdc.gov/bmi/adult-calculator/bmi-categories.html.

[39] Bowring A.L., Peeters A., Freak-Poli R., Lim M.S., Gouillou M., Hellard M. (2012). Measuring the Accuracy of Self-Reported Height and Weight in a Community-Based Sample of Young People. BMC Med. Res. Methodol..

[40] Olfert M.D., Barr M.L., Charlier C.M., Famodu O.A., Zhou W., Mathews A.E., Byrd-Bredbenner C., Colby S.E. (2018). Self-Reported vs. Measured Height, Weight, and BMI in Young Adults. Int. J. Environ. Res. Public Health.

[41] Tiggemann M., Lynch J.E. (2001). Body Image Across the Life Span in Adult Women: The Role of Self-Objectivitation. Dev. Psychol..

[42] Rojas-Guyler L., King K.A., Montieth B.A. (2008). Health-Seeking Behaviors among Latinas: Practices and Reported Difficulties in Obtaining Health Services. Am. J. Health Educ..

[43] Miller M., Michaels-Obregón A., Rocha K.O., Wong R. (2022). Imputation of Non-Response in Height and Weight in the Mexican Health and Aging Study. Real. Datos Y Espac. Rev. Int. De Estad. Y Geogr..

[44] Pérez-Salgado D., Flores J.V., Janssen I., Ortiz-Hernández L. (2012). Diagnosis and Treatment of Obesity among Mexican Adults. Obes. Facts.

[45] Winter V.R., Trout K., Harrop E., O’nEill E., Puhl R., Bartlett-Esquilant G. (2023). Women’s Refusal to Be Weighed During Healthcare Visits: Links to Body Image. Body Image.

